# Sagittal Integral Morphotype of Competitive Amateur Athletes and Its Potential Relation with Recurrent Low Back Pain

**DOI:** 10.3390/ijerph18168262

**Published:** 2021-08-04

**Authors:** Antonio Cejudo, Josep María Centenera-Centenera, Fernando Santonja-Medina

**Affiliations:** 1 Department of Physical Activity and Sport, Faculty of Sport Sciences, Regional Campus of International Excellence “Campus Mare Nostrum”, University of Murcia, 30100 Murcia, Spain; 2 Department of Surgery, Traumatology and Orthopedics, Bofill Clinic, ProActive Health, 17002 Gerona, Spain; 3 Department of Surgery, Pediatrics, Obstetrics and Gynecology, Faculty of Medicine, Regional Campus of International Excellence “Campus Mare Nostrum”, University of Murcia, 30100 Murcia, Spain; santonja@um.es

**Keywords:** back pain, risk factors, spinal deformity, injury prevention in sport, team sports

## Abstract

Athletes have higher thoracic and lumbar curvature in standing than the reference values of the non-athletic population. The sagittal integral morphotype method (SIM) assessment has not previously been applied to competitive amateur athletes (CAA). The propose of the present study was to determine the SIM of CAA treated at a sports-medicine center and to identify spinal misalignments associated with recurrent low back pain (LBP). An observational analysis was developed to describe the SIM in 94 CAA. The thoracic and lumbar curvatures of the CAA were measured in standing, sitting, and trunk forward flexion. Association analysis (Pearson’s chi-square and Cramér’s V tests) was then performed to identify the SIM misalignments associated with LBP. Effect size was analyzed based on Hedges’ g. The most common thoracic SIMs in CAA were total hyperkyphosis (male = 59.02%; female = 42.42%) and static hyperkyphosis (male = 11.48%; female = 6.06%). Hyperlordotic attitude (female = 30.30%; male = 4.92%), static-functional hyperkyphosis (male = 16.39%; female = 3.03%), and structured hyperlordosis (female = 21.21%; male = 1.64%) were the most common lumbar SIMs. Hyperlordotic attitude, static functional lumbar hyperkyphosis, and structured hyperlordosis were associated with LBP in male and female athletes.

## 1. Introduction

The sagittal thoracic and lumbar curvatures should maintain a normal range of degrees to allow for optimal static and dynamic balance, as well as proper muscle activity and body weight distribution [1,2]. These spinal curvatures are physiologically dynamic and flexible to allow for a variety of postures such as standing, trunk forward flexion, and seated trunk posture [1,3]. For example, trunk flexion reduces lumbar curvature or lordosis and increases thoracic curvature or kyphosis.

Numerous authors have reported that athletes have higher thoracic and lumbar curvature values than the reference values of the general or non-athletic population. Higher thoracic curvature has been reported in futsal players [4], soccer player [5], equestrian athletes [6], volleyball players [7], freestyle wrestlers [8], field hockey players [9], and inline hockey players [10]. In terms of lumbar curvature, soccer players [5], futsal players [4], hockey players [9], basketball players [11], inline hockey players [10], and equestrian athletes [6] had higher values than the non-athletic population. In addition, the degree of curvatures differs between sports. In this regard, the thoracic curvature of adult volleyball players [12], adolescent basketball players [13], adolescent rhythmic gymnasts [14], and the lumbar curvature of adolescent volleyball players [7] and adolescent rhythmic gymnasts [14], are significantly flatter than reported in the above studies. Most of these authors suggest that these abnormal variations in sagittal spinal curvatures (spinal misalignments) in these athletes are the result of sport-specific training schedules [15,16,17], and the predominant postures for a particular sport [11,18,19]. Heavy and repetitive training loads lead to muscle hypertrophy, muscular imbalance, asymmetry, and tightness as well as postural changes or spinal misalignments [10,15,20,21]. These spinal adaptations are more common in sports with high spinal demands [4,11,13].

Previous studies have shown associations between the spinal misalignments and spinal pathologies, and associations between spinal pathologies and back pain. Thoracic hyperkyphosis [22] and lumbar hyperlordosis [22,23,24,25] have been associated with low back pain (LBP) in non-athletes. Lumbar hyperlordosis has been correlated with LBP in female gymnasts [26], dancers [27], hockey players [28], soccer players [5,29], and basketball players [29,30,31]. Both thoracic hyperkyphosis and lumbar hyperlordosis have also been associated with spinal pathologies [32]. Thoracic hyperkyphosis has been linked to degenerative disk disease in gymnasts [33], while lumbar hyperlordosis has been linked to spondylolysis in gymnasts [34,35], dancers [36], hockey players [28], soccer players [5,29], and basketball players [30,31]. Most researchers agree that spondylolysis, spondylolisthesis, Scheuermann’s disease, and disk pathology such as lumbar disk herniation and disk degeneration are major causes of back pain in athletes of various sports [29,35,37,38,39]. With all the arguments that sport participation affects sagittal spine morphotype, it is of great interest to assess its evaluation in athletes for medical, sport, and research purposes.

The studies cited above evaluated the sagittal spine morphotype while in the standing position; however, this type of measurement does not account for spinal adaptations to other postures (sitting and trunk forward flexion). Taking this into account, Santonja et al. [40] developed a procedure to assess the sagittal spine morphotype in the most common static (standing and slump sitting) and dynamic (trunk forward flexion) positions of daily life and sports practice. The combination of these three sagittal spine morphotypes form the sagittal integral morphotype (SIM), which allows more accurate detection misalignments of the spine and facilitates the diagnosis of spinal pathologies [40]. Despite the advantages of this assessment method for the SIM, it has not been used in competitive amateur team-sport athletes (CAA).

Therefore, the present study aimed to determine the thoracic and lumbar SIM of CAA treated in a sports medicine center and to identify SIM misalignments associated with LBP. Two secondary objectives were to examine whether different types of the SIM were observed according to sex, and to determine the association between SIM misalignments and LBP in male and female athletes.

Based on the SIM evaluation, we hypothesize that structured hyperlordosis, structured lumbar kyphosis, and lumbar hypermobility are the most common lumbar SIM misalignments and are associated with recurrent LBP in CAA. In addition, male athletes have different types of SIM misalignments than female athletes. As well, the association between SIM misalignments and LBP were found in both male and female athletes.

## 2. Materials and Methods

### 2.1. Study Design

To confirm our hypothesis, an observational prospective cohort study was developed to describe the SIM and to identify sagittal spinal misalignments associated with LBP in 94 CAA. Then, an associative analysis was performed to identify SIM misalignments associated with LBP. This study was conducted in CAA who regularly participated in sports such as soccer and basketball. Athletes were asked if they volunteered to participate in this study. Thirty-four percent (*n* = 32) of CAA who participated in this study had reported a history of LBP.

All testing was performed at a sports medicine center (Clínica Bofill Center, Gerona, Spain) during the athletes’ pre-competition sports medicine examination.

The current prospective cohort considered demographic data, training regimen, anthropometric characteristics, the SIM, and data related to LBP. LBP-related data were collected over a 12-month period post the assessment baseline session of this study in the same sports medicine center. Initially, a familiarization session was conducted in which all tests included in this study were practiced. Each participant was assessed individually, and ten to twelve CAA were measured daily. The assessment baseline session included completing the questionnaire and administering the tests to determine the SIM. All CAA were asymptomatic at the assessment baseline session. Athletes were instructed not to engage in intense physical activity for 24 h prior to the assessment session, and no warm-up or stretching exercises were performed prior to testing. The medical examination room had a temperature of 25 °C. Measurements of all tests were performed simultaneously by two medical specialists in traumatology and orthopedics with over 30 years’ experience in musculoskeletal assessment. The same principal medical examiner measured the sagittal thoracic and lumbar curvatures, and the medical examiner assisted with test performance and recorded the data. The order of testing was randomized, and each test was performed three times. The average of the nearest measurements was used for data analysis. Data were then analyzed to accept or reject the null hypothesis described previously.

In a previous double-blind study (2 assessment sessions 24 h apart) with 12 young adults, the investigators demonstrated excellent intra-examiner reliability of the measurements (intraclass correlation coefficients greater than 0.90; minimum detectable change at 95% confidence less than 0.5°).

### 2.2. Participants

Ninety-four CAA from non-professional championships (soccer and basketball) took part in the study (Table 1). Their age, height, and weight were 24.35 ± 4.76 years (range: 16–30 years), 82.4 ± 11.49 kg (67.3–98.5 kg), and 1.82 ± 0.08 m (1.69–1.95 m), respectively. All CAA had more than three years of experience (8.34 ± 7.51 y) in regional competitive leagues and three training hours per week (6.52 ± 2.84 h/w). Participants had not previously received treatment for frontal or sagittal plane related pathology through the use of a brace or specific kinesiotherapy. They did not suffer from symptoms of LBP or musculoskeletal limitations to perform the tests in the assessment baseline session.

The study complied with the Code of Ethics of the World Medical Association, the Declaration of Helsinki, and was approved by the Ethics and Research Committee of the University of Murcia (Spain) for studies with human subjects (ID: 1702/2017). Participants were fully informed about the purpose and methodology of the study, and all gave their informed consent to participate in the study.

### 2.3. Self-Administered Questionnaire

CAA completed a self-administered questionnaire about their demographic, anthropometric, and sports background, systematic exercise volume data, and detailed questions about LBP (location, pain history, and severity). Questionnaire data were collected at the end the season. The information in the questionnaires was cross-checked with the assisted medical examiner. The assisted medical examiner assessed the anthropometric variables. Participants were classified into recurrent LBP, chronic LBP, or LBP-free according to LBP. Recurrent LBP consisted of episodes of LBP for less than 12 weeks. If LBP lasted more than 12 weeks or at least half of the days of the year, it was classified as chronic LBP [41,42].

### 2.4. Sagittal Integrative Morphotype Assessment Procedure

**Figure 1:**
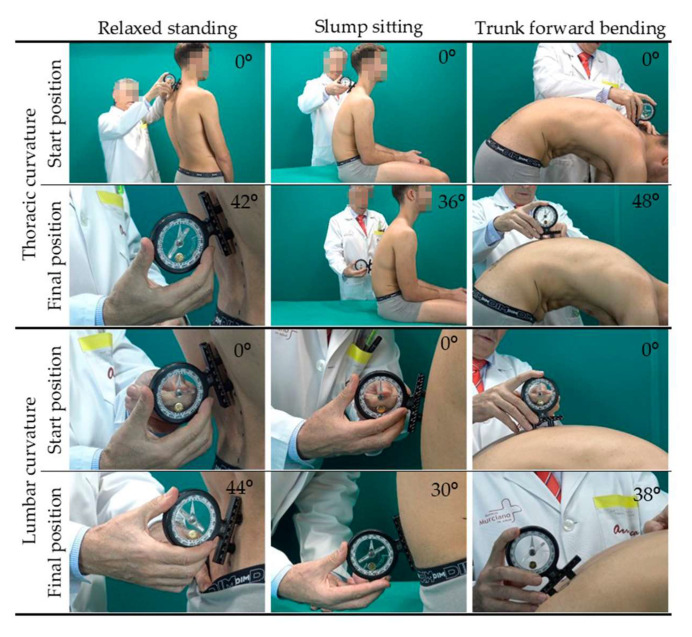
Tests for the assessment of the sagittal integral morphotype [40].

The sagittal thoracic and lumbar curvatures of the CAA were assessed in the relaxed standing position, slump sitting position, and maximum trunk forward flexion position according to the previously described procedure [27,40]. The SIM was determined by combining the sagittal spine morphotype in the three positions (Figure 1) mentioned above (relaxed standing, slump sitting, and maximum trunk forward flexion) [27,40]. Thoracic and lumbar curvatures were measured using an ISOMED Unilevel inclinometer (ISOMED, Inc, Portland, Oregon). Negative values represent the degree of posterior concavity or lordosis, and positive values represent anterior concavity or kyphosis [27].

### 2.5. Sagittal Pelvic Position Assessment Procedure

To assess sagittal pelvic alignment, the lumbo-horizontal or lumbosacral angle was measured in slump sitting position and maximum trunk forward flexion position using a goniometer following the methodology described previously [43,44].

### 2.6. Statistical Analysis

Data were exported to a spreadsheet for the initial analysis, and then SPSS v.24 (IBM, Armonk, NY, USA) was used for all analyses. A *p* < 0.05 value was used to reject the null hypothesis.

Normality of data distribution was checked using the Kolmogorov–Smirnov test. Descriptive results were reported as arithmetic means and standard deviations (SD), and gender differences in descriptive variables were calculated by independent t tests. Effect size was analyzed based on Hedges’ g (95% confidence interval). Effect size was classified as extremely large (>2.0), very large (1.00 to 2.0), large (0.6 to 1.00), moderate (0.3 to 0.6), small (0.1 to 0.3), or trivial (<0.1) according to Hopkins et al. [45].

Based on the normal ranges described by Santonja-Medina et al. [40] for the general population, the relative and absolute frequencies with normal SIM alignment or SIM misalignment were calculated for the thoracic and lumbar curvatures and the classification system of SIM. Fisher’s exact test was used to analyze the differences in the proportions of each SIM misalignment by gender. Pearson’s chi-square test and Cramér’s V-test were used to determine possible associations between SIM misalignment and recurrent LBP. Finally, Bonferroni correction analysis was considered to avoid a Type 1 error (false significance) in the results obtained for the association between SIM misalignment and recurrent LBP.

## 3. Results

Descriptive analysis suggests gender differences for the sagittal pelvic position (lumbosacral angle in slump sitting position and lumbosacral angle in maximum trunk forward flexion position), for thoracic curvature in the slump sitting position and for lumbar curvature in the relaxed standing position and in the slump sitting position (Table 1).

After interpreting the results of the curvatures assessed in each position (Table 2), thoracic hyperkyphosis was observed in the three positions (relaxed standing, slump sitting, and maximum trunk forward flexion) in a range from 54.55% to 81.97% of the participants. The percentage of participants with thoracic hyperkyphosis was higher in males (range of 73.77% to 81.97%) than in females (range of 54.55% to 63.64%). In lumbar curvature, hyperlordosis (relaxed standing position) and hyperkyphosis (slump sitting position) were observed in a range of 5.89% to 51.52% of participants. Hypokyphosis was observed in a range of 6.56% to 21.20% of participants. Female CAA showed a higher percentage of hyperkyphosis in the relaxed standing position (51.52% vs. 5.89%) and hypokyphosis in the maximum trunk forward flexion position (21.2% vs. 6.56%) than male CAA, and male CAA showed higher percentage of hyperkyphosis in the slump sitting position than the female CAA (19.67% vs. 6.06%). Significant differences between the percentage of athletes with SIM misalignments as a function of gender were only found at the slump sitting position (hyperkyphosis) for thoracic curvature (*p* = 0.0074) and at the relaxed standing position (hyperlordosis) for lumbar curvature (*p* = 0.0001).

Subsequently, the combination of the sagittal spine morphotype related to the three evaluated positions gave the thoracic SIM. Total hyperkyphosis (N = 50; male = 59.02%; female = 42.42%) and static hyperkyphosis (N = 9; male = 11.48%; female = 6.06%) were the most common misalignments of the thoracic curvature according to the SIM classification (Table 3). However, no significant differences were found between the proportions of athletes with each SIM misalignment by gender for thoracic curvature (*p* ≥ 0.136).

Table 4 shows that the most common SIM misalignments for the lumbar curvature were the hyperlordotic attitude (N = 13; female = 30.30%; male = 4.92%), static functional lumbar hyperkyphosis (N = 11; male = 16.39%; female = 3.03%), and structured hyperlordosis (N = 8; female = 21.21%; male = 1.64%). Gender differences were only found in the proportion or frequency of athletes with spinal misalignments in hyperlordotic attitude (*p* = 0.0001) and structured hyperlordosis (*p* = 0.0025).

The results of the questionnaire showed that 36 CCA had experienced LBP in the year prior to this study. After classifying the CAA into those with a history of LBP or without, no significant differences were found between the two groups on any of the variables evaluated (Table 5). However, an effect size difference is observed between the two groups according to the effect size on sport experience, and on thoracic curvature (slump sitting position) and lumbar curvature (relaxed standing and slump sitting positions).

Table 6 shows the SIM misalignments for the thoracic and lumbar curvatures in the athletes with a history of LBP.

A history of LBP was present in 38.29% (*n* = 36/94) of the athletes. All of these athletes were diagnosed with recurrent LBP. None of the athletes were diagnosed with chronic LBP. Of the 37 athletes classified with the SIM for the lumbar curvature, 23 CAA had a history of LBP (11 of 19 males and 12 of the 18 females). Analysis revealed that SIM misalignment of the lumbar curvature was associated with LBP in male (ꭕ^2^(1) = 10,690, *p* = 0.001; η² = 0.419; 57.9% of classified CAA) and female (ꭕ^2^(1) = 6347, *p* = 0.048; η² = 0.367; 66.7% of classified CAA) athletes, and both genders (ꭕ^2^(1) = 14,705, *p* = 0.000; η² = 0.396; 63.9% of classified CAA). Moreover, the magnitude of the association was found to be small in both genders (male: Cramer’s V = 0.419; *p* = 0.001; female: Cramer’s V = 0.367; *p* = 0.049; both genders: Cramer’s V = 0.396; *p* = 0.000). After Alpha correction (Bonferroni correction), an association between SIM misalignments and LBP in male (*p* = 0.0005) and female (*p* = 0.0242) athletes was observed.

## 4. Discussion

The first objective of this study was to determine the SIM of amateur athletes. The results of the presented study confirm that there are significant differences in the values of thoracic (hyperkyphosis in slump sitting position) and lumbar curvatures (hyperlordosis in relaxed standing position) between genders, which resulted in different sagittal spine morphotype profiles. These results are similar to those previously reported [10,15,17,46,47]. Indeed, male CAA in the present study had higher values of thoracic curvature and higher hyperkyphosis frequency than females in the relaxed standing position, slump sitting position, and maximum trunk forward flexion position; these results are consistent with those reported in trampoline gymnasts [17,46], artistic gymnasts [15], and the non-athletic population [48,49]. Considering these results, the male athletes of this study could have a higher influence of upper crossed syndrome. Based on Kendall [3] and Janda’s theory [50] of this syndrome, thoracic hyperkyphosis may be caused by tightness of the adductors and internal rotators of the shoulder, pectoralis minor, intercostal and abdominal muscles of the internal oblique, and weakness of the thoracic extensors and middle and lower trapezius muscles [51,52].

In the specific assessment posture of the slump sitting position, male CAA also showed a greater degree of pelvic retroversion and lumbar hyperkyphosis or inversion of lumbar curvature, respectively, in the slump sitting position. The fact that male athletes do not have adequate pelvic verticality or a normal lumbosacral angle in the slump sitting position that allows for a neutral sagittal spine morphotype in the slump sitting position was also observed in previous studies [43,44,49,53]. This inversion of the lumbar curvature is likely caused by tightness of hip extensor muscles along with weakness of the lumbar erectors and hip flexors [3]. This misalignment is commonly observed in trampoline gymnasts [17,46].

The scientific literature supports the fact that female athletes in different sports such as elite trampoline gymnasts [17,46], dressage, and show jumping riders [6] and also in the non-athletic population [48,49] have higher values of lumbar curvature in the relaxed standing position than their male counterparts. This misalignment of the lumbar can be caused by the lower crossed syndrome [3,54]. In this context, the lumbar erectors, tightness of the psoas iliacus and rectus femoris, and weakness of the abdominal muscles (especially the external oblique) and hip extensor muscles have been considered risk factors for lumbar hyperlordosis [3,55]. According to the results of this study, both male and female CAA may have the previously mentioned risk factors for thoracic and lumbar curvatures, respectively. Poor postural awareness in daily life [2,48,55] and sports [4,5,22] negatively affect the spine and promote the development of spinal misalignments.

The most common thoracic SIM misalignment in CAA was total hyperkyphosis. This is particularly severe because the athletes had hyperkyphosis in the three positions tested. Total hyperkyphosis causes increased intradiscal pressure on the anterior portion of the disc, which is greater in the maximum trunk forward flexion position and slump sitting position positions than in the relaxed standing position [56,57,58]. This process accelerates damage to the vertebral joint tissue [56,57,58]. The reported incidence of total hyperkyphosis in CAA was higher than the results reported for inline hockey players [10]. CAA’s low level of competition may be considered a risk factor for thoracic hyperkyphosis. In contrast, inline hockey players are elite competitive athletes prepared by athletic trainers, strength and conditioning specialists, and physiotherapists [10] with the aim of minimizing the risk of LBP, which is one of the most common health disorders in this sport [59]. This training program includes strengthening the core muscles, and stretching the hip and pelvic muscles [60,61]. These training requirements contribute to an adequate pelvic position and thus better sagittal spinal alignment [53]. Moreover, Moreno-Alcaraz, Cejudo and Sainz de Baranda [59] suggested, based on empirical observations, that the use of the big stick helps to prevent hyperkyphosis in the maximum trunk forward flexion position. The prevalence of total hyperkyphosis in CAA was also higher than that observed in dressage riders [6]. In equestrian sports, maintaining correct posture is essential. Judges value the elegant posture of dressage riders in dressage tests or represses to classify their level of competition [62]. In addition, proper basic rider posture promotes a more harmonious coupling between rider and horse during equitation competition and is an excellent technique for the execution of rider movements. These postural adaptations in inline hockey and equitation can compensate for other risk factors that cause total hyperkyphosis through regular exercise.

With respect to the lumbar spine SIM, significant differences by gender were observed in the proportion of athletes with hyperlordosis and structured hyperlordosis. A higher incidence was observed in females than males, and the reason for these gender differences, which have not been previously reported, is unknown. The most common type of lumbar SIM misalignment was hyperlordotic attitude with a high predominance in female CAA. Hyperlordotic attitude has been identified in previous studies assessing lumbar SIM [6,10,15]. This is defined as lumbar hyperlordosis in the relaxed standing position and normal lumbar curvature in the slump sitting position and maximum trunk forward flexion position. Regular sports practice (ice hockey, futsal, basketball, handball, volleyball, and athletics) usually causes increased lumbar curvature in SP with values above 30° [4,7,11,12,16,63]. Female CAA are likely to have weakness of the lumbar erectors and hip flexors, as well as tightness of the hip extensors, which contributes to the reduction of lumbar curvature in the relaxed standing position [3]. In this study, female CAA also showed structured hyperlordosis with higher frequency values than those previously reported in inline hockey players [10] and dressage riders [6]. The hyperlordosis in the relaxed standing position and slump sitting position and hypokyphosis in the maximum trunk forward flexion position show that the 21.21% of female CAA had structural hyperlordosis in the lumbar spine, which can cause joint tissue injury and resulting pain [25,27,64]. In male CAA, the most frequent type of lumbar SIM misalignment was the static functional lumbar hyperkyphosis, with a frequency significantly lower to those found in inline hockey players [10] and equestrian riders [6]. These athletes were characterized by lumbar hyperkyphosis in the slump sitting position and maximum trunk forward flexion position, probably due to a large pelvic retroversion caused by hamstring tightness, lumbar extensors weakness, and poor lumbopelvic rhythm [3].

The 38.29% of CAA with lumbar spinal misalignments of the SIM for lumbar curvature reported a history of LBP. The CAA with LBP showed misalignments such as hyperlordotic attitude, static functional lumbar hyperkyphosis, and structured hyperlordosis. Structured hyperlordosis and lumbar hypermobility are the most severe misalignments of the lumbar SIM classification as the misalignments are observed in the three studied positions. However, CAA with hyperlordosis and static functional lumbar spine characterized by misalignment in the relaxed standing position and slump sitting position, respectively, had a higher incidence of LBP than CAA with structured hyperlordosis and hypolordosis. Spinal positions maintained in the usual postures adopted during sports [18,19,27] and daily activities [65] have been shown to be an important risk factor for LBP. In addition, LBP history was more frequent in female CAA for all except for hypolordosis. Similar to previous studies [6,10,15,27], most of the CAA studied showed normal SIM and no LBP history.

The SIM persists and worsens with time [17,46]. For this reason, exercise programs to prevent or rehabilitate the aforementioned imbalances in CAA are needed to improve spinal alignment and consequently prevent negative consequences in the form of overload, deformity, spinal injury, and pain in CAA [66]. A multicomponent exercise program that includes flexibility, strength, and posture is necessary to improve upper and lower crossed syndrome and reduce the severity of the most common types of the sagittal spine morphotype identified in athletes with a particular focus on gender.

Future studies should add an evaluation of other risk factors for sagittal spinal misalignment and resulting low back pain such as muscle shortening and trunk weakness. In this way, Janda’s empirical theory of crossed patterns may or may not be confirmed. Similarly, a radiological examination may highlight the type of lesion causing the LBP.

## 5. Conclusions

The most common thoracic SIMs in CAA were total hyperkyphosis and static hyperkyphosis. Hyperlordotic attitude, static functional lumbar hyperkyphosis, and structural hyperlordosis were the most common lumbar SIMs in CAA. Hyperlordotic attitude, static functional lumbar hyperkyphosis, and structured hyperlordosis were associated with LBP in male and female athletes.

## Figures and Tables

**Table 1:** Data related to sports participation, sagittal pelvic position, and sagittal spinal curvatures in competitive amateur athletes.

Variables		Male (N = 61)	Female (N = 33)	ES Hedges’ g ^1^	Total ^2^ (N = 94)
Years of training		9.86 ± 7.69	5.77 ± 6.47	Moderate (g = 0.56)	8.34 ± 7.51
Training hours per week		6.52 ± 2.74	6.50 ± 3.18	Trivial (g = 0.00)	6.52 ± 2.84
LH-SSP (degrees)		103.43 ± 7.89	95.45 ± 9.17	Large (g = 0.94)	100.63 ± 9.15
LH-MTFP (degrees)		100.26 ± 13.44	86.64 ± 15.70	Large (g = 0.94)	95.48 ± 15.62
Thoracic curvature (degrees)	RSP	49.23 ± 8.15	44.94 ± 10.09	Moderate (g = 0.47)	47.72 ± 9.07
	SSP	51.92 ± 9.48	44.55 ± 9.76	Large (g = 0.76)	49.33 ± 10.16
	MTFP	73.25 ± 9.87	68.97 ± 9.90	Moderate (g = 0.42)	71.74 ± 10.04
Lumbar curvature (degrees)	RSP	32.51 ± 7.27	43.33 ± 8.24	Large (g = −1.40)	36.30 ± 9.20
	SSP	9.79 ± 8.09	1.55 ± 11.01	Large (g = 0.88)	6.89 ± 9.98
	MTFP	17.49 ± 6.62	12.94 ± 9.43	Moderate (g = 0.58)	15.89 ± 7.98

^1^ Effect size Hedges’ g; ^2^ Total: mean ± standard deviation male and female; RSP: relaxed standing position; SSP: slump sitting position; MTFP: maximum trunk forward flexion position; LH-SSP: lumbosacral angle in slump sitting position; LH-MTFP: lumbosacral angle in MTFP.

**Table 2:** Absolute and relative frequencies of 94 competitive amateur athletes within each curvature category in three positions according to normality references (Santonja-Medina et al., [40]).

Variable	Position	Category	Male (N = 61)		Female (N = 33)	
			Total (Degrees)	N (%)	Total (Degrees)	N (%)
Thoracic curvature	RSP	Hypokyphosis (<20°)	−	−	−	−
		Normal (20 to 40°)	37.50 ± 3.42	12 (19.67%)	34.67 ± 6.98	12 (36.36%)
		Hyperkyphosis (>40°)	52.10 ± 6.13	49 (80.33%)	50.81 ± 6.05	21 (63.64%)
	SSP	Hypokyphosis (<20°)	−	−	−	−
		Normal (20 to 40°)	37.55 ± 4.68	11 (18.03%)	36.67 ± 5.97	15 (45.45%) *
		Hyperkyphosis (>40°)	55.08 ± 7.00	50 (81.97%)	51.11 ± 7.03	18 (54.55%)
	MTFP	Hypokyphosis (<40°)	−	−	−	−
		Normal (40 to 65°)	60.88 ± 4.11	16 (26.23%)	59.38 ± 5.24	13 (39.39%)
		Hyperkyphosis (>65°)	77.64 ± 7.18	45 (73.77%)	75.20 ± 6.63	20 (60.61%)
Lumbar curvature	RSP	Hypolordosis (<−20°)	−18.50 ± −0.71	2 (3.28%)	−	−
		Normal (−20 to −40°)	−31.73 ± −5.13	55 (90.16%)	−36.50 ± −3.03	16 * (48.48%)
		Hyperlordotic (>−40°)	−50.00 ± −5.89	4 (5.89%)	−49.76 ± −6.05	17 (51.52%)
	SSP	Hypokyphosis (<−15°)	−18.00 ± 0.0	1 (1.64%)	−24.00 ± 5.70	2 (6.06%)
		Normal (−15 to 15°)	7.40 ± 4.43	48 (78.67%)	1.70 ± 7.08	29 (87.88%)
		Hyperkyphosis (>15°)	21.60 ± 5.40	12 (19.67%)	25.00 ± 7.09	2 (6.06%)
	MTFP	Hypokyphosis (<10°)	4.00 ± 5.89	4 (6.56%)	−0.29 ± 9.8	7 (21.2%)
		Normal (10 to 30°)	18.14 ± 5.16	56 (91.80%)	16.5 ± 5.4	26 (78.8%)
		Hyperkyphosis (>30°)	35.0 ± 0.0	1 (1.64%)	−	−

RSP: relaxed standing position; SSP: slump sitting position; MTFP: maximum trunk forward flexion position; *n*: sample size. * Significant differences between the percentage of athletes with spinals misalignments as a function of gender (*p* < 0.05).

**Table 3:** Absolute and relative frequencies of SIM (sagittal integral morphotype) misalignments for thoracic curvature in competitive amateur athletes.

Thoracic SIM Classification		Position			Male (N = 61)	Female (N = 33)
Category	Subcategory	RSP	SSP	MFTP	N (%)	N (%)
Hypokyphotic attitude	Standing	Hypokyphosis (<20°)	Normal (20–40°)	Normal (40–65°)	−	1 (1.06)
Functional hyperkyphosis	Static	Normal (20–40°)	Hyperkyphosis (>40°)	Normal (40–65°)	2 (3.28)	−
	Dynamic	Normal (20–40°)	Normal (20–40°)	Hyperkyphosis (>65)	−	2 (6.06)
	Total	Normal (20–40°)	Hyperkyphosis (>40°)	Hyperkyphosis (>65°)	5 (8.20)	2 (6.06)
Hyperkyphosis	Static	Hyperkyphosis (>40°)	Hyperkyphosis (>40°)	Normal (40–65°)	7 (11.48)	2 (6.06)
	Standing	Hyperkyphosis (>40°)	Normal (20–40°)	Normal (40–65°)	2 (3.28)	3 (9.09)
	Dynamic	Hyperkyphosis (>40°)	Normal (20–40°)	Hyperkyphosis (>65°)	4 (6.56)	2 (6.06)
	Total	Hyperkyphosis (>40°)	Hyperkyphosis (>40°)	Hyperkyphosis (>65°)	36 (59.02)	14 (42.42)

RSP: relaxed standing position; SSP: slump sitting position; MTFP: maximum flexion of the trunk position.

**Table 4:** Absolute and relative frequencies of SIM (sagittal integral morphotype) misalignments for lumbar curvature in competitive amateur athletes.

Lumbar SIM Classification		Position			Male (N = 61)	Female (N = 33)
Category	Subcategory	RSP	SSP	MFT	N (%)	N (%)
Hyperlordotic attitude	−	Hyperlordosis (>−40°)	Normal (−15 to −15°)	Normal (10 to 30°)	3 (4.92)	10 (30.30) *
Functional lumbarhyperkyphosis	Static	Normal (−20 to −40°)	Hyperkyphosis (>15°)	Normal (10–30°)	10 (16.39)	1 (3.03)
Lumbar spine with reduced mobility	Functional lumbar lordotic or Hypomobile lordosis	Normal (−20 to −40°)	Normal (0 ± 15°)	Hypokyphosis or lordosis (<10°)	2 (3.28)	−
Hypolordosis	Hypolordotic attitude	Hypolordotic (<−20°)	Normal (0 ± 15°)	Normal (10–30°)	2 (3.28)	−
Lumbar hypermobility	−	Hyperlordotic (>−40°)	Hyperkyphosis (>15°)	Hyperkyphosis (>30°)	1 (1.64)	−
Structured hyperlordosis	−	Hyperlordotic (>−40°)	Hyperlordotic (<−15°) or normal (0 ± 15°)	Hypokyphosis (<10°)	1 (1.64)	7 (21.21) *

RSP: relaxed standing position; SSP: slump sitting position; MTFP: maximum trunk forward flexion position; * Significant differences between the percentage of athletes with spinals misalignments as a function of gender (*p* < 0.05).

**Table 5:** Results of the different variables evaluated in competitive amateur athletes with a history of low back pain (LBP) or without (LBP-free).

Variables		LBP-Free (N = 58)	LBP (N = 36)	*p*-Value	ES Hedges’ g ^1^
Years of training		12.44 ± 5.72	12.62 ± 5.75	0.964	Small (g = −0.25)
Training hours per week		6.56 ± 3.12	6.46 ± 2.49	0.554	Small (g = −0.26)
LH-SSP (degrees)		100.50 ± 8.33	100.83 ± 10.46	0.433	No effect (g = −0.03)
LH-MTFP (degrees)		94.95 ± 15.47	96.33 ± 16.04	0.753	No effect (g = −0.09)
Thoracic curvature (degrees)	RSP	47.60 ± 9.36	47.92 ± 8.70	0.916	No effect (g = −0.03)
	SSP	48.67 ± 10.92	50.39 ± 8.85	0.587	Small (g = −0.17)
	MFTP	71.69 ± 9.03	71.83 ± 11.61	0.773	No effect (g = −0.01)
Lumbar curvature (degrees)	RSP	35.41 ± 8.04	37.72 ± 10.77	0.341	Small (g = −0.25)
	SSP	5.97 ± 8.22	8.39 ± 12.28	0.303	Small (g = −0.24)
	MFTP	16.14 ± 6.17	15.50 ± 10.34	0.848	No effect (g = 0.08)

^1^ Effect size Hedges’ g; RSP = relaxed standing position; SSP = slump sitting position; MFFP = trunk forward flexion position; LH-SSP: lumbosacral angle in slump sitting position; LH-MTFP: lumbosacral angle in trunk forward flexion position.

**Table 6:** Classification of SIM (sagittal integral morphotype) misalignments of the thoracic and lumbar curvatures of amateur competitive athletes with a history of LBP (N = 36).

**Classification SIM for Thoracic Curvature**		**Position**			**Low Back Pain**	
**Category**	**Subcategory**	**SP**	**SSP**	**MFT**	**Male (N = 61)**	**Female (N = 33)**
Hyperkyphosis	Standing	Hyperkyphosis (>40°)	Normal (20–40°)	Normal (40–65°)	0	1
Functional hyperkyphosis	Dynamic	Normal (20–40°)	Normal (20–40°)	Hyperkyphosis (>65)	0	2
	Total	Normal (20–40°)	Hyperkyphosis (>40°)	Hyperkyphosis (>65°)	3	0
Hyperkyphosis	Dynamic	Hyperkyphosis (>40°)	Normal (20–40°)	Hyperkyphosis (>65°)	0	2
	Total	Hyperkyphosis (>40°)	Hyperkyphosis (>40°)	Hyperkyphosis (>65°)	12	8
**Classification SIM for Lumbar Curvature**		**Position**			**Low Back Pain**	
**Category**	**Subcategory**	**RSP**	**SSP**	**MFTP**	**Male (N = 61)**	**Female (N = 33)**
Hyperlordotic attitude	−	Hyperlordosis (>−40)	Normal (−15 to −15°)	Normal (10 to 30°)	1	7
Functional lumbar hyperkyphosis	Static	Normal (−20 to −40°)	Hyperkyphosis (>15°)	Normal (10–30°)	7	1
Structured hyperlordotic	−	Hyperlordosis (>−40°)	Hyperlordosis (<−15°) or normal (0 ± 15°)	Lordosis or Hypokyphosis (<10°)	1	4
Hypolordotic attitude	−	Hypolordosis (>−20°)	Normal (−15 to −15°)	Normal (10 to 30°)	2	0

RSP = relaxed standing position; SSP = slump sitting position; MTFP = maximum trunk forward flexion position.

## Data Availability

The data sets used and analyzed during the current study are available from the first or corresponding author on reasonable request.
